# An association of adult personality with prenatal and early postnatal growth: the EPQ lie-scale

**DOI:** 10.1186/2050-7283-2-8

**Published:** 2014-03-31

**Authors:** Trine Flensborg-Madsen, Rasmus Revsbech, Holger Jelling Sørensen, Erik Lykke Mortensen

**Affiliations:** Unit of Medical Psychology, Institute of Public Health, University of Copenhagen, Øster Farimagsgade 5, 1353 Copenhagen K, Denmark; Hvidovre Psychiatric Center, Dep. 807, Cognitive Research Unit, Brondbyostervej 160, 2605 Brondby, Denmark; Mental Health Centre Copenhagen, Bispebjerg Bakke 23, 2400 Copenhagen NV, Denmark; Center for Healthy Aging, Faculty of Health and Medical Sciences, University of Copenhagen, Blegdamsvej 3B, 2200 Copenhagen K, Denmark; Institute of Preventive Medicine, Frederiksberg Hospital, Hovedvejen 5, Nordre Fasanvej 57, 2000 Frederiksberg, Denmark

**Keywords:** Eysenck personality questionnaire, Lie-scale, Extraversion, Neuroticism, Psychoticism, Birth weight, Birth length, Birth head-circumference

## Abstract

**Background:**

Recent studies have noted differences in social acquiescence and interpersonal relations among adults born preterm or with very low birth weight compared to full term adults. In addition, birth weight has been observed to be negatively correlated with lie-scale scores in two studies. We attempted to replicate and extend these studies by examining young adult lie-scale scores in a Danish birth cohort.

**Method:**

Weight, length and head circumference of 9125 children from the Copenhagen Perinatal Cohort were measured at birth and at 1, 3 and 6 years. A subsample comprising 1182 individuals participated in a follow-up at 20–34 years and was administered the Eysenck Personality Questionnaire (EPQ) which includes a lie-scale (indicating social acquiescence or self-insight). Associations between lie-scale scores and weight, length and head circumference respectively were analysed by multiple linear regression adjusting for single-mother status, parity, mother’s age, father’s age, parental social status, age at EPQ measurement, intelligence, and adult size.

**Results:**

Male infants with lower weight, length, and head-circumference at birth and the following three years grew up to have higher scores on the lie-scale as young adults. Most of these associations remained significant after adjustment for the included covariates. No associations were found for females. Analyses were also conducted with neuroticism, extraversion and psychoticism as outcome variables, but no significant associations were found for these traits after adjustment.

**Conclusions:**

The findings replicate and extend findings from previous studies suggesting that size at birth and during the first three years of life is significantly associated with social acquiescence in adult men. They highlight the potential influence of prenatal and early postnatal development on personality growth and development.

## Background

While pre- and postnatal suboptimal growth has convincingly been demonstrated to be associated with *physical health* risks such as cardiovascular diseases (Barker 2004; Barker et al. 2005, 2009; Roseboom et al. 2006) and type 2 diabetes (Barker 2004; Barker et al. 2009; Lawlor et al. 2006; Roseboom et al. 2006), another line of evidence suggests that suboptimal growth is also related to *mental health* in general. Hence, suboptimal pre- and postnatal growth may also predict risk of schizophrenia (Abel et al. 2010; Byrne et al. 2007; Cannon et al. 2002; Nilsson et al. 2005; Perrin et al. 2007; Wahlbeck et al. 2001), personality disorders (Fazel et al. 2012; Lahti et al. 2010; Monfils et al. 2009), depression (Anderson et al. 2006; Brown et al. 2000; Raikkonen et al. 2007, 2008), and anxiety (Anderson et al. 2006; Levyshiff et al. 1994; Somhovd et al. 2012) as well as low adult intelligence (Eriksen et al. 2010; Richards et al. 2001, 2002). These findings are in line with the Developmental Origins of Health and Disease (DOHaD) hypothesis which suggests that a suboptimal early life environment may permanently alter developing organ structures and the functionality of biological systems and thus result in increased risk for diseases later in life (Barker 2004).

In line with studies of psychiatric disorders, recent findings have focused on associations between personality traits and pre- and postnatal growth. Thus, preterm birth and very low birth weight have proven to be associated with higher neuroticism and agreeableness in addition to lower extraversion (Hertz et al. 2013), difficulties in establishing social contacts (Hille et al. 2008), antisocial behaviours (Hack et al. 2004), cautiousness (Waxman et al. 2013), and to be more likely to be cautious, shy, risk aversive and less extraverted (Schmidt et al. 2008). In representative samples, research of associations between birth weight and personality traits is sparse and based on either The Helsinki Birth Cohort Study 1934–44 (HBCS) or the English national child development study (NCDS) from 1958. In HBCS, a recent study found low birth weight to be associated with neuroticism in late adulthood (61–63 years), such that individuals born with a low birth weight (≤2500 g) had higher neuroticism scores compared to those weighing more at birth. In addition, they found height and weight growth trajectories from birth to adulthood to be associated with extraversion (Lahti et al. 2013). Other studies, based on the HBCS found that, birth weight, length, and head circumference were associated with cognitive abilities, temperament, hostility, trait anxiety, depression, and ADHD symptoms later in life (Lahti et al. 2008; Raikkonen et al. 2007, 2008; Raikkonen and Pesonen 2009). Similarly, NCDS found mental distress to be inversely associated with birth weight (Cheung et al. 2002).

Lie scales were originally introduced into personality measures in order to detect the “faking good” of scores on other scales (O’Donovan 1969). It has, however, been suggested that lie scales in general should be interpreted as measuring a personality dimension in its own right (Furnham 1986; McCrae and Costa 1983). According to Eysenck & Eysenck (1976), the lie scale included in the Eysenck Personality Questionnaire (EPQ) permits lying to be diagnosed when a set of rarely performed desirable acts are endorsed as being typical of the respondent, and when common non-desirable acts are subsequently denied. The unitary nature of the Eysenckian lie scale has been questioned, and more than one distinct personality component has been suggested (Francis 1991; Francis et al. 1991; Pearson and Francis 1989). According to some researchers, the dimension is best characterized as *social acquiescence or conformity* (Birenbaum and Montag 1989; Massey 1980) and according to others as (lack of) *self-insight* (Crookes and Buckley 1976; Francis et al. 1983; Francis 1991; Kirton 1977).

In addition to the studies concerning social acquiescence mentioned above, *two* studies have investigated specifically whether size at birth is associated with lie-scale scores in adulthood. A study by Allin et al. (2006) found that very preterm males scored higher on the lie-scale (although this did not quite reach statistical significance), and that birth weight in general was negatively correlated with lie-scale score. In accordance with these results, Schmidt et al. found extremely low birth weight to be associated with higher shyness and lower sociability, and lower birth weight, overall, to be associated with higher lie-scale scores (Schmidt et al. 2008).

In this study, we will attempt to replicate and extend these findings by examining whether high lie-scale scores in adulthood are associated with smaller size at birth. We had opportunity to investigate this question in the Copenhagen Perinatal Cohort (CPC) which includes measures of weight, length, and head-circumference at birth and during the first six years as well as young adult lie-scale scores measured by the EPQ (Eysenck and Eysenck 1975). Since repeated measures of size during the first 6 years were available, it was possible to analyse, not only associations with prenatal growth, but also associations between size in early childhood and young adult lie-scale scores. Stronger associations of personality with birth weight may suggest that prenatal growth may influence personality development, whereas an age-related increase in the strength would suggest that postnatal growth plays a more relevant role.

## Methods

### Study population

The study objectives were investigated using data from the Copenhagen Perinatal Cohort (CPC) and from a follow-up study of this birth cohort, the Prenatal Development Project (PDP). *The Copenhagen Perinatal Cohort* was initially established with data on 8,949 mothers and their 9,125 consecutive deliveries born at the University Hospital in Copenhagen during the period 1959–61. Information on demographic, socioeconomic, prenatal, and postnatal factors were recorded prospectively during pregnancy, at delivery, and at a 1-year examination (Zachau-Christiansen and Ross 1975). The mothers were mainly residents in Copenhagen, but some were admitted on obstetrical complications or because of single mother status (Villumsen 1970). A total of 8,400 infants survived the first month after birth. A subsample comprising 1575 members of the Perinatal Cohort were selected on the basis of pre- and perinatal records and 1249 participated in *The Prenatal Development Project* between 1982 to 1994 (Reinisch et al. 1993). The full test battery included a 2–4 hour home assessment by a social worker and an 8–11 hour psychological evaluation conducted at the Institute of Preventive Medicine (Reinisch et al. 1993; Mortensen et al. 2002). Several personality and cognitive tests were administered, including the Eysenck Personality Questionnaire (EPQ) (Eysenck and Eysenck 1975) which was administered to 1182 participants.

### Early weight, length, and head circumference

Birth weight (kg), length (cm) and head circumference (cm) were measured immediately after birth by either a midwife or a nurse and again at 1, 3 and 6 years (Villumsen 1970).

### Adult follow-up

The adult follow-up took place when the participants were 20.3–34.5 years old (SD = 4.31). Among other measures, the comprehensive assessment included the following:

#### Lie-scale scores

Eysenck Personality Questionnaire (EPQ) developed in 1975 (Eysenck and Eysenck 1975) was used. The Danish version comprises 101 binary “yes” or “no” questions from which scores on the personality traits of extraversion, neuroticism, psychoticism and lie-scale are derived (21 questions are included in the lie-scale).

#### Physical measurements

Weight (kg), length (cm), and head circumference (cm) were obtained as part of a comprehensive assessment of physical characteristics.

#### Intelligence

The complete Wechsler’s Adult Intelligence Scale (WAIS) was administered (Wechsler 1958), and the Full Scale IQ is included in the presented analyses.

### Statistical analyses

The missing data rate on weight, length, and head circumference between the ages of 0 and 6 years varied from 2.8% to 49.5% with missing data rates generally increasing with increasing age of the child. Participants with incomplete information on exposure variables were excluded from the analysis in which the particular exposure was analysed. T-tests were used to evaluate associations between birth weight and potential confounding factors and mediators (Table 1).

**Table 1:** **Descriptive characteristics of possible confounding factors**

Exposure variables^a^	Mean level of birthweight	
	Men (P-value^b^)	Women (P-value^b^)
**Single mother**		
**Yes**	3213 g (0.14)	3191 g (0.90)
**No**	3312 g	3183 g
**Parity (first parity)**		
**Yes**	3220 g (0.004)	3166 g (0.58)
**No**	3353 g	3190 g
**Mother’s age**		
**< 24.5**	3261 g (0.30)	3179 g (0.99)
**≥24.5**	3314 g	3178 g
**Father’s age**		
**<28.5**	3273 g (0.47)	3159 g (0.47)
**≥28.5**	3308 g	3193 g
**Parental social status**		
**Lower end (<4)**	3275 g (0.68)	3127 g (0.12)
**Higher end (≥4)**	3297 g	3204 g
**Age of EPQ measurement**		
**≤29 years**	3320 g (0.22)	3176 g (0.67)
**>29 years**	3261 g	3195 g
**Adult intelligence**		
**< 103**	3257 g (0.22)	3133 g (0.05)
**≥ 103**	3315 g	3222 g
**Adult weight**		
**≤ 68.5**	3074 g (<0.0001)	3146 g (0.001)
**>68.5**	3356 g	3318 g
**Adult height**		
**≤ 173**	2995 g (<0.0001)	3156 g (0.001)
**>173**	3342 g	3374 g
**Adult head-circumference**		
**≤ 56.5**	3116 g (<0.0001)	3153 g (0.001)
**>56.5**	3337 g	3357 g
**Birth-length**		
**< 51 cm**	2761 g (<0.0001)	2790 g (<0.0001)
**≥51 cm**	3553 g	3496 g
**Head circumference, birth**		
**< 34.5 cm**	2835 g (<0.0001)	2948 g (<0.0001)
**≥34.5 cm**	3551 g	3526 g

^a^Exposure variables are divided according to 50% distribution.

^**b**^Two independent samples t-test.

Estimates of the associations between weight, length, and head circumference during the first six years and personality traits in adulthood were computed by means of multiple linear regression models (Rothman 1998) adjusting for: Single-mother status, parity, mother’s age, father’s age, parental social status, and age of EPQ measurement. These variables were chosen based on the literature in addition to theoretical considerations of potentially confounding factors; they were not dichotomized in the regression models but included as continuous variables. Missing values of the covariates varied from 0.08% (parity) and 0.25% (mother’s age) to 10.7% (parental social status).

To evaluate the independent contribution of size at 1, 3 and 6 years, previous growth variables (i.e. birth weight and weight at 1 year in analyses of weight at 3 years) were included as covariates in a separate analysis. In several other studies, intelligence in adulthood has been found to be associated with size at birth (Eriksen et al. 2010; Richards et al. 2001, 2002), and this was also the case in the present study where intelligence was significantly associated with both birth weight and with lie-scale scores. Thus, it is possible that associations between early physical characteristics and lie-scale scores are mediated by intelligence. It is also possible that the associations between early physical characteristics and lie-scale scores are mediated by adult physical characteristics. Consequently, supplementary analyses were conducted including adult intelligence and adult physical characteristics as covariates in the fully adjusted model (i.e. adult weight in analyses of weight at 0, 1, 3 and 6 years, etc.) in order to evaluate these estimates (Tables 2 and 3).

**Table 2:** **MEN: Beta weights for childhood measures of weight, length and head circumference in regression models predicting adult lie-scale scores**

Exposure variable	N	Unadjusted b	Adjusted^a^b	Fully adjusted – including previous growth^b^b	Fully adjusted - including IQ^c^b	Fully adjusted - including adult size^d^b	Imputed values (40 datasets), adjusted^a^b
Birth weight (kg)	578	-0. 64**	-0. 52	-	-0.47	-0.48	-0.57*
Weight 1 y (kg)	432	-0.52**	-0.43**	-0.37*	-0.41**	-0.47**	-0.46***
Weight 3 y (kg)	347	-0.26*	-0.22*	-0.007	-0.18	-0.30**	-0.24**
Weight 6 y (kg)	285	0.01	-0.02	0.12	-0.02	-0.06	-0.004
Birth length (cm)	573	-0.14*	-0.13*	-	-0.13*	-0.08	-0.13*
Length 1 y (cm)	438	-0.13*	-0.11*	-0.09	-0.11*	-0.03	-0.11*
Length 3 y (cm)	358	-0.11*	-0.11*	-0.09	-0.09	-0.06	-0.10*
Length 6 y (cm)	299	-0.05	-0.07	-0.03	-0.07	-0.04	-0.04
Head circumference birth (cm)	534	-0.30**	-0.25*	-	-0.22*	-0.29**	-0.25**
Head circumference 1 y (cm)	533	-0.19	-0.17	-0.12	-0.13	-0.23	-0.18
Head circumference 3 y (cm)	358	-0.26*	-0.26*	-0.10	-0.21	-0.41**	-0.20
Head circumference 6 y (cm)	298	-0.25	-0.19	0.02	-0.11	-0.26	-0.10

a: Single-mother status, parity, mother’s age, father’s age, parental social status, and age of EPQ measurement.

b: Single-mother status, parity, mother’s age, father’s age, parental social status, age of EPQ measurement, and previous growth (i.e. birth weight and weight at 1 year in analyses of weight at 3 years).

c: Single-mother status, parity, mother’s age, father’s age, parental social status, age of EPQ measurement, and adult intelligence.

d: Single-mother status, parity, mother’s age, father’s age, parental social status, age of EPQ measurement, and adult size (i.e. adult weight in analyses of weight at 0, 1, 3 and 6 years etc.).

*P ≤ 0.05; **P ≤ 0.01; ***P ≤ 0.001.

**Table 3:** **WOMEN: Beta weights for childhood measures of weight, length and head circumference in regression models predicting adult lie-scale scores**

Exposure variable	N	Unadjusted b	Adjusted^a^b	Fully adjusted – including previous growth^b^b	Fully adjusted - including IQ^c^b	Fully adjusted - including adult size^d^b	Imputed values (40 datasets), adjusted^a^b
Birth weight (kg)	571	-0.19	-0.31	-	-0.24	-0.42	-0. 08
Weight 1 y (kg)	428	0.08	0.08	0.17	0.15	0.04	0.03
Weight 3 y (kg)	337	0.001	-0.03	0.003	0.02	-0.08	-0.02
Weight 6 y (kg)	292	0.03	0.02	0.19	0.05	0.04	0.07
Birth length (cm)	569	0.02	-0.03	-	-0.02	0.005	0.04
Length 1 y (cm)	425	-0.01	-0.009	-0.0007	0.007	0.03	-0.008
Length 3 y (cm)	342	-0.01	-0.03	-0.04	-0.02	-0.03	-0.02
Length 6 y (cm)	300	-0.01	-0.02	0.03	0.002	-0.01	-0.003
Head circumference birth (cm)	523	0.05	0.07	-	0.09	0.09	0.02
Head circumference 1 y (cm)	427	-0.02	-0.005	0.09	0.05	0.07	0.03
Head circumference 3 y (cm)	340	-0.17	-0.11	0.07	-0.05	-0.22	-0.07
Head circumference 6 y (cm)	299	-0.004	0.04	0.21	0.09	0.06	0.06

a: Single-mother status, parity, mother’s age, father’s age, parental social status, and age of EPQ measurement.

b: Single-mother status, parity, mother’s age, father’s age, parental social status, age of EPQ measurement, and previous growth (i.e. birth weight and weight at 1 year in analyses of weight at 3 years).

c: Single-mother status, parity, mother’s age, father’s age, parental social status, age of EPQ measurement, and adult intelligence.

d: Single-mother status, parity, mother’s age, father’s age, parental social status, age of EPQ measurement, and adult size (i.e. adult weight in analyses of weight at 0, 1, 3 and 6 years etc.).

*P ≤ 0.05; **P ≤ 0.01; ***P ≤ 0.001.

Different models were used for inclusion of confounding and mediating factors (See Tables 2 and 3). All analyses were stratified according to sex because preliminary analyses revealed significant interactions between sex and physical size variables on the EPQ Lie Scale (weight at 1 year, birth length, and birth head circumference).

To evaluate the influence of missing data on the results, supplementary analyses were conducted using multiple imputation in which missing values on weight, length and head-circumference were replaced with imputed values (generated from values of weight, length and head-circumferences, the lie-scale score and covariates) and analysed using 40 imputed datasets (Schafer 1997). Adjusted linear regression results are shown for the multiple imputation analyses (Tables 2 and 3).

The assumption of normal distribution of residuals of the lie-scale score was evaluated graphically and only showed minor deviations which should not bias estimates or standard-errors (Wooldridge 2006). The assumption of linearity was evaluated graphically and showed no deviations. Additionally, linearity of regression was analysed by testing the significance of a quadratic component for all exposure variables. This component was not found significant for any of the exposure variables (except for weight and length at 3 years *as predictors of neuroticism*, where the quadratic component explained 0.57% and 0.60% respectively).

All statistical analyses were conducted by means of the statistical software package SAS 9.1.

In addition to investigating lie-scale scores, all above-mentioned analyses were conducted for neuroticism, extraversion and psychoticism as measured in the EPQ.

## Results

Table 1 shows associations between the covariates and mean levels of birth weight. Adult weight, adult height, adult head-circumference, birth-length, and head-circumference at birth were all significantly associated with the mean level of birth weight for both genders. In addition, parity was associated with birth weight for men, while adult intelligence was associated with birth weight for women.

In the regressions analyses, several significant associations were found in men. Hence, lower weight, smaller length, and smaller head-circumference at birth, at 1 year, and at 3 years were generally associated with higher scores on the lie-scale in adulthood. Most associations remained significant after inclusion of all covariates; for example the adjusted regression coefficient for weight at 1 year was -0.43 (p < 0.01), while it was -0.11 (p < 0.05) for length at 1 year (Table 2). Adjusting for previous growth, the estimates generally became smaller and in most cases non-significant.

For males, inclusion of adult intelligence as covariate did not change the overall pattern of results, although weight, length, and head circumference at age 3 were no longer significantly associated with lie-scale scores. When adjusting for adult measures of weight and head-circumference, the estimates for weight at the ages of 1 and 3 years and head-circumference at birth and at 3 years increased. The estimates for length generally became smaller and non-significant when adjusting for adult height. Thus, it is possible that adult height may mediate the associations between early physical growth and size and adult personality. Imputing missing values did not change the adjusted estimates noteworthily.

No significant associations with physical size were found for the lie-scale in women (Table 3).

Extraversion, neuroticism and psychoticism showed few significant associations with weight, length and head circumference during the first six years: For *extraversion*, significant associations were found only for women with weight at 1 year (b = -0.39; p ≤ 0.05) and 6 years (b = -0.22; p ≤ 0.01), but after adjustment for previous growth these estimates became non-significant. For *neuroticism*, no significant associations were found for either men or women for any of the exposure variables. Associations with *psychoticism* were found only for women where weak associations were observed for birth weight and birth length. These associations, however, became non-significant after adjustment for covariates. (Data not shown). The lie-scale was significantly correlated with extraversion (r = -0.16; p < 0.0001), psychoticism (r = -0.27; p < 0.0001), and intelligence (r = -0.20; p < 0.0001) (Data not shown).

## Discussion

### Main results

Our analyses reveal associations between small size at birth, at 1 year and at 3 years of age, and a higher score on the EPQ lie-scale in adulthood. Male infants with lower weight, length, and head-circumference at birth and the following three years grew up to have higher scores on the lie-scale as young adults.

### Methodological issues

The main advantage of this study is the prospective design including real-time documentation of weight, length and head-circumference repeated over a period of six years. Nevertheless, the frequency of missing data tended to be high especially for measures at the 3 and 6 year assessments. A further limitation of the study is that Type I error is a possibility, as we conducted tests on 12 measures of weight, length and head-circumference. However, the fact that the lie-scale was consistently associated with weight, length and head-circumference, in addition to the fact that none of the other EPQ scales showed significant associations, points towards a consistent pattern and thus a non-incidental finding.

As in all observational studies, there may be unrecognized confounding factors associated with size and lie-scale scores. Single-mother status, parity, mother’s age, father’s age, parental social status, age at EPQ measurement, intelligence, and adult size were considered confounding or mediating factors in the linear regression models. However, there may be other unobserved confounding factors influencing both physical growth and brain development (see: “Interpretation of findings”). These factors were not included in the analyses as appropriate data were not available, and therefore we do not know the impact of these factors on the associations at hand.

### Comparison with other studies

The most comparable findings from previous studies are: (1) The study by Allin et al. (2006) who found that birth weight was negatively correlated with the lie-scale (r = -0.23; P = 0.051) in their sample of 108 adults with a history of very preterm birth. In addition, they found that males born very preterm did not score significantly different from full term males on the extraversion, neuroticism, and psychoticism scales, which is in accordance with our findings of no significant associations between physical size and these traits. Interestingly, the authors also found that smaller head circumference at 18 to 19 years was associated with increased lie scale scores (Allin et al. 2006). (2) The study by Schmidt et al. (2008) who reported that in their extremely low birth weight sample of 71 young adults, lower birth weight was associated with higher shyness, behavioural inhibition, loneliness and lie-scale scores (r = -0.26; P < 0.01). Hence, our results as well as the cited studies suggest that measures of weight and of head circumference seem to be the most important predictors of adult lie-scale scores – both of which are commonly assumed to be relatively valid indicators of brain maturation during the last weeks of gestation and during the first postnatal years (Cooke et al. 1977; Haukvik et al. 2013; O’Connell et al. 1965).

Other studies support the two studies by pointing towards low birth weight or preterm birth as being associated with social acquiescence and interpersonal relations, such as difficulties in establishing social contacts (Hille et al. 2008), antisocial behaviours (Hack et al. 2004), cautiousness (Waxman et al. 2013), agreeableness (Hertz et al. 2013), neuroticism (Lahti et al. 2013; Hertz et al. 2013), and higher extraversion (Hertz et al. 2013), in addition to being more likely to be cautious, shy, risk aversive and less extraverted (Schmidt et al. 2008).

### Interpretation of findings

The inverse association between size during the first three years and lie-scale scores in adulthood might be explained by several biological or psychological mechanisms. *A biological explanation* is that physical growth and size may be associated with development of brain structures, which influence later development of personality traits related to lie-scale scores. Hence, specific genes or environmental factors (such as early parenting or psychosocial stress) may contribute to size at birth and growth in childhood in addition to affecting the overall development of the brain and thereby the development of personality. Previous studies suggest that possible characteristics in the brain associated with lie-scale scores are striatal D_2/3_ receptor availability which in several studies have been shown to be negatively correlated with lie-scale scores (Egerton et al. 2010; Huang et al. 2006; Reeves et al. 2007). Biological explanations of prenatal factors are supported by our results showing the associations to be strongest for size at birth and to be diluted later in childhood where the estimates generally became smaller and in most cases non-significant for both weight, length and head circumference when adjusting for previous growth. Examples of such prenatal factors are malnutrition (de Rooij et al. 2012) or smoking (Ekblad et al. 2010) during pregnancy, pregnancy complications or maternal stress during pregnancy (Weinstock 2005), which in addition to predicting differences in birth size may also predict personality in the offspring. Finally, epigenetic changes in gene expression as a consequence of prenatal (or early postnatal) size (Turan et al. 2012) could be of importance.

*A psychological explanation* is that the experience of being large or small is an important factor in the causal network leading to a high lie-scale score. A recent study found that babies down to the age of 8 months were able to perceive social dominance or hierarchy on the basis of mere physical size (Thomsen et al. 2011) suggesting that children very early in life experience an association between social hierarchies and physical dominance. Thus size, even in the very early years, may influence the development of self and self-insight (which is sometimes considered the trait primarily measured by the lie-scale), and the association between size and lie-scale scores may reflect a causal link. This explanation, however, suggests that the effects of size would increase in later childhood which was not confirmed by our findings of stronger associations for the early measures of size.

Due to our insufficient knowledge of the traits assessed by the lie scale, the developmental trajectories of these traits and the factors influencing thems, it is not possible to reach conclusions concerning biological and psychological explanations, and the interpretation of the associations between early size and the traits assessed by the lie scale remains an open question.

## Conclusions

The results suggest that scores on the EPQ lie-scale are associated with weight, length, and head-circumference at birth and the following three years in men, while no significant associations were found for the lie-scale in women. Although the mechanisms underlying these associations remain largely unidentified, the findings highlight the potential influence of prenatal and early postnatal development on personality growth and development.

As this is the first study to investigate scores on the lie-scale in relation to early physical growth and size, more research should be conducted in the area to elucidate the present findings. In particular, the psychological conceptualization of the lie-scale should be further evaluated in empirical studies, and in addition, further studies of possible confounding and mediating factors should be conducted to better evaluate the causal network leading to empirical associations between early physical growth and size and scores on the lie scale.
